# Reversing Acute Cardiomyopathy With Coenzyme Q10 Supplementation in Cobalamin B Disease: A Case Report and Literature Review

**DOI:** 10.1002/jmd2.70063

**Published:** 2025-12-25

**Authors:** Dalia Said, Aisha Al Shamsi

**Affiliations:** ^1^ Genetic Metabolic Division, Pediatrics Department Tawam Hospital Al Ain UAE

**Keywords:** cardiomyopathy, cobalamin B disease, coenzyme Q10, heart failure, methylmalonic acidemia

## Abstract

Methylmalonic acidemia (MMA) is a rare metabolic disorder with various subtypes, including Cobalamin B (cblB) disease. While cardiac complications are well‐documented in propionic acidemia, their occurrence in MMA is less understood. Here, we report a 12‐year‐old child with cblB disorder who developed acute cardiomyopathy (CM). The patient presented with fever, tachycardia, and dyspnea. Echocardiography revealed depressed left ventricular function, which initially improved with standard treatment but rapidly deteriorated. Coenzyme Q10 (CoQ10) supplementation was initiated at a dose of 25 mg/kg/day, resulting in a remarkable improvement in cardiac function within 72 h. This report highlights the potential efficacy of CoQ10 in treating MMA‐related cardiomyopathy, suggesting mitochondrial dysfunction as a key factor. The successful use of CoQ10 in this context is novel, as previous literature mainly focused on its application in propionic acidemia. This report proposes CoQ10 supplementation as a promising adjuvant therapy for cardiomyopathy in MMA, particularly in cblB disorder, and calls for further research to validate its benefits.

## Key Points/Highlights

- Acute cardiomyopathy (CM) can occur in methylmalonic acidemia (MMA), including cobalamin B (cblB) subtype, even in the absence of metabolic decompensation.
- High‐dose Coenzyme Q10 (25 mg/kg/day) can lead to rapid and complete recovery of left ventricular function as in our patient.
- The dramatic response supports secondary mitochondrial dysfunction and functional CoQ10 deficiency as a key mechanism in MMA‐related CM.
- Early empirical CoQ10 supplementation should be considered as an adjunctive therapy in MMA‐associated CM.

## 1 Introduction

Methylmalonic acidemia (MMA) is an autosomal recessive organic acidemia caused by deficient methylmalonyl‐CoA mutase (MCM) activity or impaired transport and synthesis of its cofactor, cobalamin. In particular, cobalamin B (cblB) disease, a subtype of MMA, results in a defect in the ATP:cobalamin adenosyltransferase, the enzyme responsible for converting cobalamin into AdoCbl. Deficiency in this enzyme leads to failure in AdoCbl synthesis, impairing MCM function and causing the accumulation of toxic metabolites such as methylmalonic acid. This disease has a broad spectrum of clinical manifestations, the most significant of which is nervous system impairment, as well as other multiple organ systems. Recently, cardiovascular involvement has begun to draw attention as an increasing number of cardiac complications have been reported [1].

Cardiomyopathy has been recognized as a common complication of PA. Both dilated and hypertrophic cardiomyopathy have been observed. The reported prevalence ranges from 7% to 39% in PA cohorts [2]. Arrhythmias or cardiomyopathy (dilated or hypertrophic) have been reported in 10%–20% of individuals with isolated MMA, primarily *mut* ^*0*^ or *mut‐* (and *cblB* subtypes, as well as in the B_12_‐responsive *cblA* subtype) [3].

Liver transplantation (LT) has been proposed as a therapeutic measure for heart failure due to MMA or PA, with reported improvements or stabilization of left ventricular ejection fraction, particularly in PA. However, improvement is not universal and recurrence or progression of cardiomyopathy after transplantation has been reported.

Successful cases of combined heart and LT and isolated heart transplantation were documented in patients with PA. To the best of our knowledge, cardiomyopathy in a patient with MMA post liver transplant has not been reported or it may be extremely rare. It is noteworthy to remind readers that liver and heart transplants are expensive medical procedures, involving advanced surgical techniques and long‐term post‐operative care. The success of these transplants is further complicated by the limited availability of suitable donors, which often leads to extended waiting periods. These factors contribute to the overall complexity and cost of the procedures, making them challenging for both patients and healthcare systems [4].

In 2011, Prada et al. reported three patients with MMA and sudden onset CM in the setting of previous and ongoing metabolic crises. Unfortunately, all patients died due to the rapidly deteriorating cardiac condition. The first patient was 22 years old, known to have MMA at 4 months old, presented with a 10‐day history of nausea and non‐bloody/non‐bilious emesis. The second patient, who was diagnosed with MMA since day 2 of life, presented at 7 months of life with an upper respiratory infection, vomiting, and hyperammonemia, with an Echo revealing dilated cardiomyopathy (DCM) with LVEF of 27% necessitating the initiation of anti‐failure medication. He died at the age of 2. The third patient, who was diagnosed in the neonatal period and had frequent metabolic decompensations, ultimately developed DCM with an LVEF of 20%–25% and died at 4 years of age [5].

It is well established that Coenzyme Q10 (CoQ10) is crucial for mitochondrial electron transport and ATP synthesis, and it serves as a key antioxidant in cardiac tissue. In MMA, methylmalonic acid accumulation may induce cellular CoQ10 deficiency and impair mitochondrial respiratory chain activity, potentially contributing to cardiomyopathy [6]. Multiple systematic reviews and meta‐analyses indicate that CoQ10 supplementation in heart failure may modestly improve left ventricular ejection fraction and is associated with possible reductions in all‐cause mortality and heart failure hospitalizations without evidence of increased adverse outcomes. However, the quality of the evidence is low to moderate, trial sizes are limited, and the findings are not specifically tailored to MMA [7]. The American Heart Association notes that while small studies and meta‐analyses suggest modest benefits, larger randomized trials are needed, and the clinical value of this approach remains uncertain [8].

Here, we report a patient with MMA clbB subtype who presented with CM and we propose using CoQ10 to improve and perhaps reverse cardiac function.

## 2 Case Description

A 12‐year‐old female who was diagnosed to have MMA of the cblB complementation type since birth by biochemical testing, complementation analysis, and molecular testing including the *MMAB* gene sequencing, followed by whole exome sequencing (WES) in 2022 was done and showed only a pathogenic homozygous splice site c.197‐1G>T, exon 3 (NM_052845.3) variant in the *MMAB* gene. She is a product of healthy, consanguineous parents with two healthy siblings. Her antenatal course was smooth and uncomplicated. She was born at term by an emergency C‐section due to breech presentation. Initially, she required no resuscitation. At few hours old, she started to be lethargic with poor feeding and hypothermia. She was found to have a low glucose level of 40 mg/dL (2.2 mmol/L); therefore, she was shifted to the Neonatal Intensive Care unit (NICU) and started treatment for neonatal sepsis. During her stay in NICU, she was found to have persistent metabolic acidosis requiring frequent sodium bicarbonate; she remained hypoactive, so serum ammonia was measured and found to be 370 micromol/L/L. Sodium benzoate was initiated. Metabolic workup, including urine organic acid profile and acylcarnitine profile, was suggestive of a biochemical diagnosis of MMA, so she was started on hydroxycobalamin injection. However, she showed a minimal response and persistent high methylmalonic acid levels. She spent about 1 month in the NICU. She was discharged home on carnitine, sodium benzoate, and NG tube feeding with disease‐specific formula (Propimex 1—Methionine and Valine free, low in Threonine and Isoleucine).

During her routine follow‐up, she remained relatively well, with few admissions for acute illness to prevent any metabolic decompensation. At 8 years of age, she had repeated urinary tract infections with worsening of renal function. A renal biopsy revealed tubulointerstitial nephritis and tubular atrophy, and she was labeled to have chronic kidney disease (stage III). Her annual Echo, held every year since the initial echo at 7 months of age, was reported as normal. On cardiology follow‐up at the age of 11 years, she was found to have a prolonged QT interval with normal cardiac function and structure, and she was maintained on Nadolol.

At 12 years of age, she presented to the emergency department with fever, tachycardia, and dyspnea. Her glucose was normal at 7.1 mmol/L, ammonia was 53.5 micromol/L, blood gas showed high anion gap metabolic acidosis (as baseline secondary to chronic kidney disease), and urine ketones were negative. Hence, metabolic crisis/decompensation was ruled out. She also complained of abdominal pain and was more comfortable sitting upright, leaning forward. Therefore, pancreatic enzymes were done to rule out acute pancreatitis in the setting of her underlying MMA, and came back normal. With her unstable vitals: temperature 39.2°C, HR 122 bpm, Resp 40–50/min, SPO2 95%, cardiac workup was done, which revealed markedly elevated cardiac enzymes (Table 1). Chest X‐ray was unremarkable. Urgent echocardiography showed mildly depressed LV function (52%–54%), with septal dyskinesia; the posterior wall was moving well, with a normal collapsing IVC and normal RV function. The diagnosis of cardiomyopathy (CM) was made. She was admitted to the Pediatric Intensive Care Unit (PICU). She was maintained at a high glucose concentration (25% dextrose) with restriction to 2/3 maintenance and milrinone infusion, maintaining her antiarrhythmic medication Nadolol. As calories are essential to prevent her from metabolic decompensation, she was initiated on Intralipid along with intravenous fluid. Appropriate monitoring of blood glucose, ammonia, blood gases, and urine ketones was performed. Her baseline methylmalonic acid level at this admission was elevated (3702 nmol/mL). No infectious cause was identified, as her viral MDx (influenza, adenovirus, rhinovirus, parainfluenza, and human metapneumovirus) was negative, including COVID‐19 PCR, except for elevated SARS‐CoV‐2 antibodies (241.00 units/mL), which may indicate past infection. She was already on carnitine supplementation (62 mg/kg/day), and her total and free carnitine were elevated; despite that, carnitine was increased to the maximum of 3 g/day as per emergency protocol in metabolic crisis.

**TABLE 1:** Investigations.

	On admission	Follow‐up ~10 months	Reference range
Complete blood count	WBC 10.8 × 10^9^/L (PMN 8.78 × 10^9^/L) RBC 4.49 × 10^12^/L Hgb 117 g/L Plts 323 × 10^9^/L	WBC 10.7 × 10^9^/L (PMN 7.28 × 10^9^/L) RBC 3.73 × 10^12^/L Hgb 94 g/L Plts 360 × 10^9^/L	WBC 4.5–13.5 × 10^9^/L RBC 3.8–5.0 × 10^12^/L Hgb 11.5–15.5 g/L Plts 140–400 × 10^9^/L
Electrolytes	Na 133 mmol/L, K 4.3 mmol/L, Cl 93 mmol/L, CO_2_ 93 mmol/L	Na 141 mmol/L, K4.9 mmol/L, Cl 97 mmol/L, CO_2_ 32 mmol/L	Na 136–145 K 3.2–5.5 Cl 98–107 CO_2_ 22–29
Creatinine	105 micromol/L (high)	115 micromol/L (high)	34–77
Urea	9.95 mmol/L (high)	8.50 mmol/L (high)	2.8–8.1
Blood gas	pH 7.37, pCO_2_ 45.2 mmHg, HCO_3_ 26 mmol/L, lactate 2.0 mmol/L	pH 7.44, pCO_2_ 49.9 mmHg, HCO_3_ 34 mmol/L, lactate 12.0 mmol/L	pH 7.35–7.45, pCO_2_ 35–45, HCO_3_ 22–26, lactate 0.5–2.2
Ammonia	53.5 micromol/L	34.8 micromol/L	15–51 micromol/L
CRP	19.3 mg/L	8.5 mg/L	< 5 mg/L
Pro‐BNP	> 35000.0 ng/L ng/L	8537.0 ng/L	0–186 ng/L
Troponin	63.7 ng/L	23.1 ng/L	< 14 ng/L
Total CK	227 IU/L	48 IU/L	26–192 IU/L
Calcium	2.46 mmol/L	2.44 mmol/L	2.1–2.6 mmol/L
Amylase	86 units/L	74 units/L	28–100 units/L
Lipase	16 IU/L	28 IU/L (Normal)	13–60 units/L
Liver enzymes and function	AST 24 IU/L ALT 6 IU/L Albumin 32 g/L	AST 82 IU/L ALT 160 IU/L Albumin 28 g/L	AST < 32 IU/L ALT < 33 IU/L Albumin 35–52 g/L
Total carnitine level	252 nmol/mL	302 nmol/mL	34–77 nmol/mL
Methylmalonic acid, quantitative, plasma	3702 nmol/mL	1153 nmol/mL, After single B12 injection	≤ 0.40 nmol/mL

**FIGURE 1:**
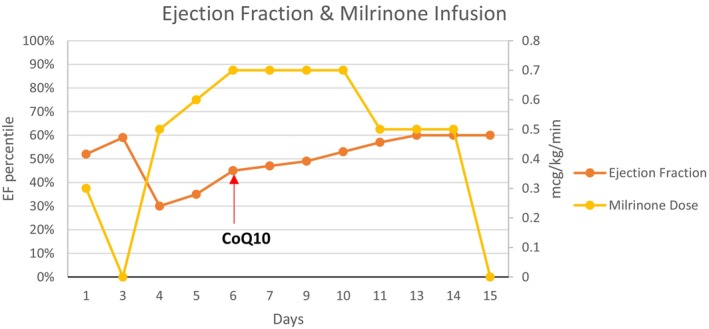
Ejection fraction and milrinone infusion throughout treatment. Red arrow marks the commencement of Coenzyme Q10 (CoQ10) on Day 6 of clinical course.

After 48 h of admission, her echocardiography showed improvement, so milrinone was gradually weaned off. However, within 24 h, she relapsed again with moderate to severe LV dysfunction with EF 30%–35%, dilated IVC and minimal collapsing, normal RV function, and a bilateral small amount of pleural effusion. At this stage, it was suggested that CoQ10, which can help with CM, be started, so she was commenced on 200 mg orally q6 hours (equivalent to 25 mg/kg/day). Daily Echocardiography was reassuring, and within 72 h, her cardiac function was restored to normal. She successfully weaned off milrinone and was discharged home on CoQ10 in addition to her other chronic medications (Figure 1).

## 3 Discussion

**FIGURE 2:**
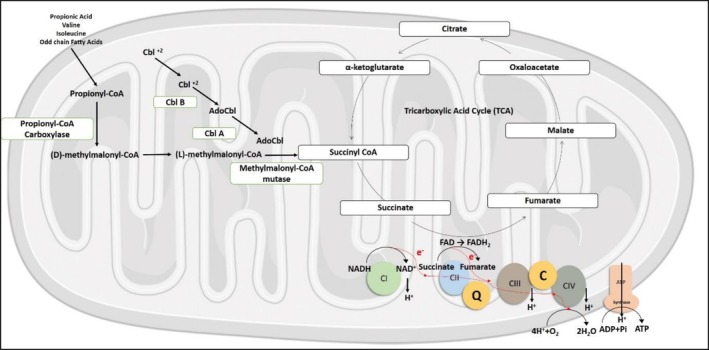
Methylmalonic acidemia (MMA), which may result from impaired function of methylmalonyl‐CoA mutase (MCM) or impaired intracellular cobalamin (Cbl) transport and processing. This leads to the accumulation of methylmalonyl‐CoA and further accumulation of propionyl‐CoA. Methylcitrate is formed by the condensation of propionyl‐CoA with oxaloacetate. This leads to the lack of substrates for the tricarboxylic acid cycle (e.g., oxaloacetate, citrate, and α‐ketoglutarate), further compromising the synthesis of NADH and FADH2 as electron donors to the respiratory chain. In particular, Complex 2 (CII) of the respiratory chain is affected, leading to overall mitochondrial dysfunction.

CoQ10 or ubiquinone is a lipid‐soluble and biologically active molecule localized in the inner mitochondrial membrane (IMM), with main functions as an electron carrier in the electron transport chain, maintenance of the proton gradient crossing IMM (Figure 2), as well as acting as a lipophilic antioxidant [9, 10].

According to Proctor et al., who described the human neuronal cell CoQ10 status and mitochondrial function, and stated that in organic acidemias such as PA and MMA, cellular toxicity is thought to result from synergistic inhibition of the respiratory chain and the tricarboxylic acid (TCA) cycle by propionyl‐CoA and its alternative products, in particular 2‐methylcitrate and malonate. A deficiency of CoQ10 could further aggravate mitochondrial energy metabolism impairment. The accumulation of toxic metabolites has been reported to increase lipid and protein peroxidation and decrease the activity of antioxidant enzymes [6]. The accumulation of propionyl‐CoA in mitochondria may be necessary but not sufficient for the development of cardiomyopathy. In this secondary mitochondriopathy, other factors that promote its development may depend on stress, such as the immune system perturbing cardiomyocyte gene expression and promoting cardiac remodeling [4]. A failing heart is in a state of insufficient energy due to defects in oxidative phosphorylation of fatty acids, while anaerobic glycolysis is enhanced [11, 12].

Pu et al. demonstrated that mice models with dilated cardiomyopathy (DCM) showed lower myocardial content of CoQ10 in mouse hearts starting from the early phase of DCM and becoming more severe with time. Furthermore, genes for CoQ10 biosynthesis enzymes were identified and almost all were downregulated [10]. The use of CoQ10 for PA‐associated CM has been reported by Baruteau et al. [12]. Two siblings with propionic acidemia (PA) were reported, with the older sibling initially showing a mild condition managed with standard treatments, including a low‐protein diet and carnitine supplementation. At age 15 years, a mildly dilated left ventricle was observed, and by age 17 years, he developed severe decompensated DCM after weeks of lethargy and poor feeding, accompanied by mild metabolic decompensation and hyperammonemia (161 μmol/L). No infectious cause was identified. During ventricular assist implantation, a cardiac biopsy revealed endocardial fibrosis, enlarged mitochondria, and reduced myocardial CoQ10 levels. Despite various metabolic and cardiac support therapies, the condition worsened, prompting a significant increase in CoQ10 dosage from 1.5 to 25 mg/kg/day when the low level of myocardial CoQ10 was determined. Over time, heart function improved, leading to the eventual removal of mechanical support after 67 days.

Considering the severity of myocardial CoQ10 depletion and the increasing evidence of secondary mitochondrial impairment as one of the main pathophysiological explanations for cardiomyopathy in PA, we thought that CoQ10 supplementation should be considered as an adjuvant therapy in PA‐related cardiomyopathy [12].

It is worth noting that in the organic acidemias, PA and MMA, cardiomyopathy arises from interlinked mitochondrial dysfunction and other metabolic derangements, exacerbated by conditions such as thiamine deficiency. In PA, metabolite by‐products (e.g., propionyl‐CoA, methylcitrate) suppress mitochondrial enzymes such as pyruvate dehydrogenase and α‐ketoglutarate dehydrogenase, which corrupt the TCA cycle and ATP synthesis, causing defective cardiac energy metabolism. Similarly, MMA leads to mitochondrial electron transport chain dysfunction due to the accumulation of methylmalonic acid, causing oxidative stress and cardiac disease.

## 4 Conclusion

To the best of our knowledge, the use of CoQ10 in CM has been reported only in patients with PA, and most studies on liver and heart transplants for CM have also been associated with PA. There is no literature suggesting the use of CoQ10 for CM in patients with MMA, making this case report unique. This report highlights the promising hypothesis regarding the responsiveness of MMA‐related CM to CoQ10, suggesting that secondary mitochondrial dysfunction may be a key contributing factor. We strongly recommend CoQ10 supplementation as an adjuvant therapy for CM in MMA and perhaps to be established as standard of care since the time of diagnosis. Even when the CoQ10 level is unknown, initiation of supplementation is advisable. This is because the serum level may not accurately reflect severe CoQ10 depletion in critical tissues, such as the heart. Further controlled studies are required to validate efficacy, determine optimal dosing, and assess safety in this population.

## Author Contributions

Authors contributed to the conception and design. All authors contributed to the acquisition and revision of the manuscript and agreed to be accountable for all aspects of the work, ensuring integrity and accuracy. All authors read and approved the final manuscript.

## Funding

The authors have nothing to report.

## Consent

Consent for publication was obtained from the reported patient's parents.

## Conflicts of Interest

The authors declare no conflicts of interest.

## Data Availability

The data that support the findings of this study are available on request from the corresponding author. The data are not publicly available due to privacy or ethical restrictions.
